# Supplementary material for: The CovR regulatory network drives the evolution of Group B Streptococcus virulence
Source: PLoS Genet. 2021 Sep 7;17(9):e1009761. doi: 10.1371/journal.pgen.1009761 (PMC8448333; doi:10.1371/journal.pgen.1009761)
Supplement: Attachment — Submitted filename: Response to reviewer.pdf [file pgen.1009761.s018.pdf]

Dear Editors,

We thank the reviewers for their positive evaluation and for their comments to improve the manuscript.
Please find below our responses to all the points raised by the reviewers.
The main comments were answered by:

a) *the complementation of the two mutants on the chromosome and their validation by RT-qPCR. In addition to the reviewer's suggestion, we also constructed new mutants (by swapping the mutation) to validate the specificities of the mutants (new Fig. 1D and 1E).*
b) *the sequencing of the mutant's chromosomes (new supplementary Table S12)*
c) *an improved characterization of the epitope-tagged system by Western (new supplementary figure S 1G) and RT-qPCR against the native protein (new supplementary figure S 1F).*

Responses to the Reviewers' Comments:

Reviewer #1: The study by Mazzuoli and colleagues is a useful addition to the large amount of previously-generated data with regard to the importance of the CovR/S regulatory system in the virulence of GBS. This study uses powerful, genome-wide technologies to characterize molecular explanations for the variation in the CovR regulons previously observed between GBS isolates. The combining of RNA-Seq and ChIP-Seq datasets was particularly powerful, and has provided new insights into the specifics of CovR-mediated regulation (e.g. direct vs indirect regulation). For the most part, the experiments performed we done so in scientifically-sound manners, were explained sufficiently such that they could be repeated by others, and the resultant data are presented in engaging and informative manners. While I am excited about the topic as a whole, there are some issues that I believe should be addressed regarding specific aspects of the research:

Major
• Given the highly-sensitive comparisons done with the mutant strains, particularly the transcriptome comparisons between the ΔcovR and covR-D53A mutant strains, it needs to be confirmed that these strains harbor no spurious mutations. This can be achieved by complementing each strain with covR (inserted into the chromosome so that there are no gene-dosage consequences) and repeating the RNA-Seq to show that both complemented strains are identical to the parental (WT) strain. One of several alternatives, and probably an easier approach at this stage, would be for you to perform WGS on the two covR mutants and confirm that the covR mutations are the only ones in the genome that distinguish them from the WT strain. This should also be done with the strains shown in Figure 5.

As suggested, we have complemented the two mutants (Δ*covR* and *covR* D_{53}A) on the chromosome (by restoring a wild-type allele at the *covR* locus, resulting in the complemented strains denoted Δ->WT and D_{53}A->WT). In addition to the reviewer suggestion, we have 'swapped' the mutations, meaning we have generated a Δ*covR* mutant in a D_{53}A background (D_{53}A->Δ) and, reciprocally, a D_{53}A mutant in a Δ*covR* background (Δ->D_{53}A).

We confirmed the complementation for 10 genes in the D_{53}A->WT and Δ->WT strains by RT-qPCR. In addition, the swapped mutants (D_{53}A->Δ and Δ->D_{53}A) nicely confirmed the up-regulation of four of the selected genes in the Δ*covR* mutants only. These new RT-qPCR results have been included in the new Fig. 1D and 1E and are described in the first result section.

New Fig1D and 1E =

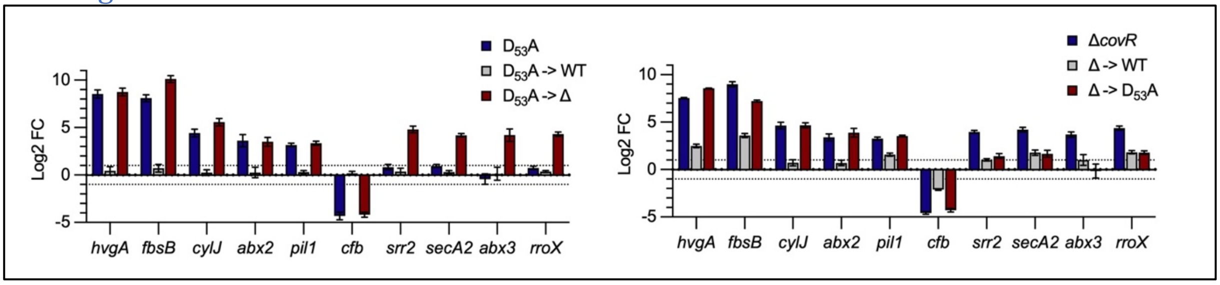

(D) Validation of gene expression by qRT-PCR in the $CovR_{D53A}$ mutant (D_{53}A), the chromosomally complemented strain (D_{53}A->WT), and in a Δ*covR* mutant done in the $CovR_{D53A}$ background (D_{53}A->Δ).
(E) Validation of gene expression by qRT-PCR in the Δ*covR* mutant (Δ), the chromosomally complemented strain (Δ->WT), and in a $CovR_{D53A}$ mutant done in the Δ*covR* background (Δ->D_{53}A). Means and standard deviations of log2 fold change (mutant versus WT) are calculated from three biological replicates.
N.B : the initial Fig. 1D have been moved to the supplementary figure S2A.

We also fully sequenced the genomes of the mutants (including those of the newly generated complemented and swapped strains). The results are summarized in a new supplementary table S12. In the BM110 background, one and three secondary mutations are present in the chromosome of the D_{53}A and Δ*covR* mutants, respectively. Additional secondary mutations (1 to 4) are present in the other sequenced mutants. Although secondary mutations are common during GBS mutant construction, the nature of the mutations suggested that the inactivation of *covR* might be associated with compensatory mutations (probably linked to the over-expression of metabolic genes, such as ABC transporters, directly regulated by CovR). These secondary mutations result in a partial complementation in the Δ*covR* background but do not have an effect in the D_{53}A background or in the swapped mutants, confirming the specificities of the Δ*covR* and D_{53}A mutants (Fig. 1D and 1E).

• In the strains in which FLAG-CovR is induced, what is the concentration of CovR in the cells? How does this compare to CovR levels in the WT strain? The simplest way to look at this would be to use an anti-CovR antibody to do a side-by-side Western. I worry that, due to the placing of CovR on a multicopy plasmid, that the level of CovR is not physiologically-relevant….which would have a big impact on the global binding characteristics and therefore on the relevance of the ChIP-Seq data (too little, and only the high-affinity promoters will be bound, too much, and off-site binding may occur).
After several attempts, we finally obtained a suitable anti-CovR antibody and we compared the level of CovR expression in the BM110 wild-type strain with the level of the episomal FLAG-CovR variant by Western. As shown in the new supplementary figure S1 panel G, the conditions used for ChiP-seq (50 and 200 ng/ml aTc) correspond to a low and a near WT level of CovR.
New S1G panel =

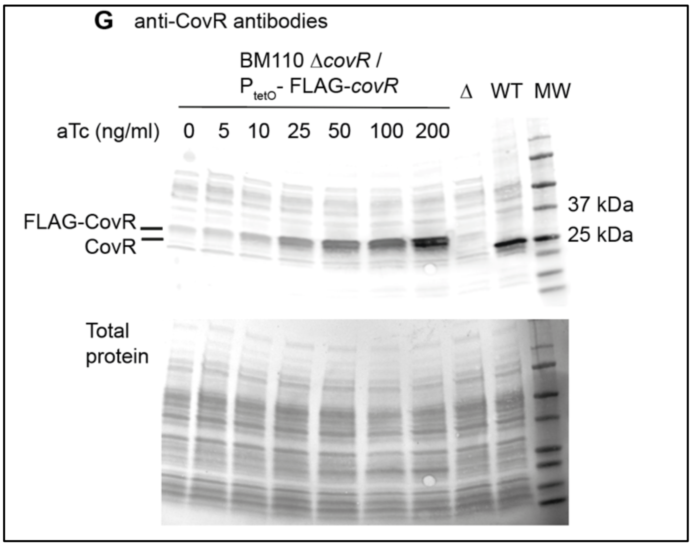

(G) Comparative analysis of CovR and FLAG-CovR expression with anti-CovR antibodies. Western were done with 20 µg of total protein extracts of the BM110 WT, of the Δ*covR* mutant, and of the Δ*covR* / pTCV-P_{tetO}-FLAG-*covR* mutant grown with increasing concentration of aTc (0 – 200 ng/ml). Membranes were hybridized with anti-CovR antibodies and revealed with fluorescent secondary antibodies (upper panel). Loading controls are given by the non-specific hybridization signal (upper panel) and by Ponceau S coloration (bottom panel).

• I see no data that confirms that the addition of the FLAG-tag to CovR does not alter the regulatory activity of the protein. If it does, then this negatively impacts the data gained from the use of this strain. Placing the FLAG-tag into the chromosomally-encoded covR gene in the parental strain (and not via a multi-copy plasmid….due to gene dosage concerns…..which are particularly pronounced and worrisome for regulatory genes) and showing that this strain is identical to the parental strain for the mRNA levels of a range (i.e. some with high-affinity CovR promoters and some low-affinity) of regulated genes would be one way to do this.

To test if the FLAG-epitope alters the regulatory activity of CovR, we compared by RT-qPCR the repression of 5 selected genes by the WT *covR* or the FLAG-*covR* cloned in the same expression system (the pTCV-P_{tetO} vector). As shown in the new supplementary Fig. S1 panel F, similar repressions are observed with the two vectors (considering the different level of *covR* transcription due to the highly sensitive anhydrotetracycline-dependent induction generating experimental variability, especially at the lowest concentrations). This indeed confirms that the expression level of a regulatory gene is critical but shows that the FLAG-epitope has no significant impact on the functionality of CovR in this system.

New S1F panel =

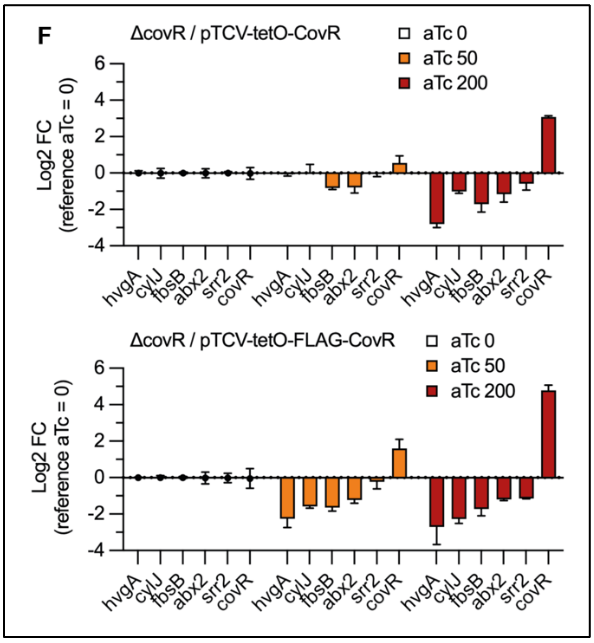

(F) Comparative analysis of transcriptional repression by *covR* (upper panel) and FLAG-*covR* (bottom panel) cloned into the same pTCV-P_{tetO} inducible vector into the BM110 Δ*covR* mutant. RT-qPCR were done starting with RNAs prepared from uninduced (aTc 0 ng/ml) and induced (aTC 50 and 200 ng/ml) cultures with two biological replicates with technical triplicates.

• Fig 1B shows hvgA being regulated by CovR with a fold-change log2 value of 7, while Fig S1D shows that this gene only has a fold-change of a log2 value of 2.5-3 following the induction of covR. What accounts for this discrepancy? It should be discussed.

As pointed by the previous comment, difference in gene dosage or in the transcriptional level of a regulatory gene is critical. And, indeed, our inducible ectopic expression system does not fully complement the Δ*covR* mutant (either with a WT CovR or a FLAG-CovR). Nevertheless, the ectopic expression of FLAG-CovR is functional (Fig S1) allowing using it for ChIP-sequencing.
The episomal expression of an epitope-tagged variant has some limitations. Yet, it has been extensively used (including systematically in M. tuberculosis: e.g.: Nature. 2013 doi: 10.1038/nature12337 / Nature Comm. 2015 doi: 10.1038/ncomms6829) and compared favorably with the gold standard, which is the immunoprecipitation of the native protein with highly selective antibodies (e.g. as done for the M. tuberculosis PhoP regulator: PLoS Path 2014 doi: 10.1371/journal.ppat.1004183).

• To significantly strengthen the hypothesis that there are differences in the CovR regulon between CC17 and CC23 strains additional isolates (e.g. 3-5) of each CC should be investigated. This could be, for example, via qRT-PCR of select mRNAs. Otherwise, the described differences between BM110 and NEM316 may simply be strain-specific.

Indeed, we discussed the representativeness of the two strains in the original manuscript (BM110 is likely representative of the CC-17 complex clonal but NEM316 is likely not representative of the whole non-CC17 GBS population). We are currently characterizing this diversity of *covR* regulation at the population level, which is beyond the scope of this manuscript.

Minor

• There appears to be a discrepancy between figures 1B and 1E. In 1B, it looks like srr2 is

differentially-regulated at a higher rate in the ΔcovR mutant than the D53A mutant, but the opposite is true in figure 1E. I believe the labeling is mixed up for figure 1E?

Thank you for pointing out this error. We have substituted this figure with the new RT-qPCR dataset, which includes srr2 and 3 additional genes showing an overexpression in the Δ*covR* backgrounds only (new Fig. 1D and 1E).

• I don't know if binding of non-phosphorylated CovR to DNA has been shown in GBS, but I know it has for GAS. Either way, I would add a reference at the end of line 132 that highlights this for GBS (or GAS if no GBS example is known).

As suggested, we added references for the *S. pyogenes* CovR orthologue as well for other response regulators.

• To expand access to this work for people outside of the GBS field, I would add a little more info (1 or 2 sentences) about the bibA/hvgA (line 58) and srr1/srr2 stories (line 59).

As suggested, we added a short description on the HvgA/BibA and Srr1/Srr2 relationships.

• To enhance viewing and interpretation, I suggest adding a faded diagonal line (bottom left to top right) to figures 1B and 5C that would highlight identity between the strains.

As suggested, the former Supp. Fig S6B (identity between BM110 and NEM316 genomes) has been moved in Fig. 5A.

• What are the values listed on the Y-axis of the graphs in figure S2B?

The Y-axis values are normalized reads counts. We have added the legend on the graph.

Reviewer #2: The CovRS TCS of S. agalactiae (Group B Strep, GBS) is a critical sensory system responsible for expression modulation of roughly 10-15% of the genome, including genes critical in infection. Although the CovRS system has been thoroughly studied in S. pyogenes and several strains of GBS, genome analysis of the CovR regulon has not been described for clonal complex CC-17, which is most highly associated with late-onset meningitis in neonates that is disseminated globally—it is a critical group to be analyzed. Several fundamental questions surrounding the CovRS regulatory system remain unanswered and include determining which genes are under its control in CC-17, and which of these are under CovR's direct regulation. The present study utilizes RNA-sequencing and ChIP-sequencing studies on two types of CovR loss-of-function mutants (a covR deletion and a D53A point mutant unable to undergo phosphorylation). All experimental methodologies are sound are and rigorously conducted.

Primary findings:
1. The manuscript's primary source of data presents the genetic regulon of CovR in CC-17 strain BM110 and informs on CovR binding sites across the genome. The effort placed into confirming DNA binding specificity and location is substantial and of extraordinarily high quality. Figures 1-3 and suppl. Figures S1-S3 all provide excellent content.
We thank the reviewer for his/her comment.

2. Indirect and secondary regulation by CovR is described thoroughly and with appropriate discussion. Suggestion: possibly consider citing this original observation of N-NS in salmonella (DOI: 10.1126/science.1128794).

As suggested, we cited the seminal observation in the discussion.

3. The manuscript's most important concept, that CovR-regulated genes are disproportionately under positive selective pressure, is evaluated by genome-wide analysis of mutation frequencies comparing CovR-regulated and unregulated genes, as well as comparisons to a non-CC-17 strain. Bolstering the conclusions that the CovRS regulon is highly plastic and varied between isolates are demonstrations that kinase/phosphatase activities are strikingly different between BM110 and NEM316. Describing differences in the gene regulon, and differences in kinase/phosphatase activities between strains is not necessarily a new concept (especially in literature describing S. pyogenes CovRS regulation); however, the present report make an explicit case that the regulatory system governs the most critical gene sets that account for intra-species evolution and host adaptation. While these conclusions might require a deeper dive into genome comparisons to flesh out rates of variation, it is my sense that the presented data are sufficient to make an important contribution to field.
We thank the reviewer for his/her comment. Indeed, based on the results presented in this manuscript, we are currently exploring the diversity of CovR regulation in several GBS clones to more accurately describe the variation in the population.

Other suggestions
1. Fig. 2D. Identification of the binding site is interesting, important, and matches very well to what has been documented in S.pyogenes. However, there has been some controversy over this in the pyogens literature (please seehttps://doi.org/10.1046/j.1365-2958.2002.02810.x compared to https://doi.org/10.1038/srep12057). Perhaps this should be mentioned in the results or discussion.
We mentioned this point in the result section, including by citing the recent *S.* pyogenes genome-wide analysis (https://doi: 10.1128/mBio.01642-21).

2. Line 142. Should state explicitly what is represented by 'peaks' (sites where CovR binds).
We clarified what we meant by peaks ('the distribution of sequencing reads mapped on the chromosome have the typical characteristics of a ChIP-seq signal, forming a peak centered on or near the regulator binding site').

3. Line 201. It is not clear what is meant by, "the functions encode by groups 1 and 2 genes did not differ from the function encode by genes of the direct regulon."
We clarified this point ('Next, we asked whether the functions encoded by the extended regulon differ from those of the direct regulon, but both encode for similar functions mainly associated with virulence and metabolites transport (Table 2 and Supplementary Table S2B)').

4. Line 233, it was unclear to me which categories of figure 4 that the "24 genes associated with CovR binding accumulated more mutations than expected…"
Indeed, Fig. 4 summarized the supplementary table S4 (the 24 genes corresponding to column 'ORF mutational biases in the population). In the initial Fig. 4, we spitted this group into several categories (mutational biases with or without additional intergenic mutational biases and CC-17 specific or not).

We have simplified Fig. 4 with only 3 categories and have better referenced the additional table S4 in the text. We have also moved the former Supp Fig. S4 into Fig. 4 to illustrate difference in the *fbsA* regulation and to highlight promoter mutations between the two WT strains.

5. Is anything known about what CovS responds to? Again, given the close parallels, I think it would benefit a reader to know that in S. pyogenes that LL-37 and Mg2+ are modulators of CovS kinase/phosphatase activity. Perhaps the paragraph at line 396 would be an ideal place to discuss this.
CovS in *S. agalactiae* does not respond to LL-37 and Mg2+ (P. Trieu-Cuot unpublished observations). We added a sentence in the discussion.
